# Effort-reward imbalance and work ability: cross-sectional and longitudinal findings from the Second German Sociomedical Panel of Employees

**DOI:** 10.1186/1471-2458-12-875

**Published:** 2012-10-15

**Authors:** Matthias Bethge, Friedrich Michael Radoschewski, Christoph Gutenbrunner

**Affiliations:** 1 Department for Rehabilitation Medicine, Coordination Centre for Applied Rehabilitation Research, Hannover Medical School, Hannover, Germany; 2 Department of Health Care Research and Quality Management in Rehabilitation, Charité - Universitätsmedizin Berlin, Berlin, Germany

## Abstract

**Background:**

Although data from longitudinal studies are sparse, effort-reward imbalance (ERI) seems to affect work ability. However, the potential pathway from restricted work ability to ERI must also be considered. Therefore, the aim of our study was to analyse cross-sectional and longitudinal associations between ERI and work ability and vice versa.

**Methods:**

Data come from the Second German Sociomedical Panel of Employees. Logistic regression models were estimated to determine cross-sectional and longitudinal associations. The sample used to predict new cases of poor or moderate work ability was restricted to cases with good or excellent work ability at baseline. The sample used to predict new cases of ERI was restricted to persons without ERI at baseline.

**Results:**

The cross-sectional analysis included 1501 full-time employed persons. The longitudinal analyses considered 600 participants with good or excellent baseline work ability and 666 participants without baseline ERI, respectively. After adjustment for socio-demographic variables, health-related behaviour and factors of the work environment, ERI was cross-sectionally associated with poor or moderate work ability (OR = 1.980; 95% CI: 1.428 to 2.747). Longitudinally, persons with ERI had 2.1 times higher odds of poor or moderate work ability after one year (OR = 2.093; 95% CI: 1.047 to 4.183). Conversely, persons with poor or moderate work ability had 2.6 times higher odds of an ERI after one year (OR = 2.573; 95% CI: 1.314 to 5.041).

**Conclusions:**

Interventions that enable workers to cope with ERI or address indicators of ERI directly could promote the maintenance of work ability. Integration management programmes for persons with poor work ability should also consider their psychosocial demands.

## Background

The rapid growth of transnational interdependence of capital, trade and labour that characterises economic globalisation seems to be changing work and employment. These changes go along with work intensification, job insecurity, poor quality of work and wage inequalities in a highly competitive labour market [1-4]. One model that covers these aspects of work is the effort-reward imbalance (ERI) model [5]. The ERI model is based on the equity theory and the notion of contractual reciprocity. According to Siegrist [5], the beneficial effects of employment (participation, self-efficacy and approval) depend on fairness in the relationship between employer and employee. Most importantly, employees expect adequate rewards (income, esteem, career opportunities and job security) for their efforts. The ERI model therefore assumes that a lack of reciprocity (i.e. high effort and low reward) results in emotional distress and adverse health effects.

Cumulative evidence from high-quality cohort studies and meta-analyses support the hypothesis that ERI contributes to adverse health effects, in particular, coronary heart diseases and mental disorders [6-10]. ERI is also linked to increased sick leave, intention to retire and exit from paid employment [11-13]. Recent studies also provide evidence that ERI is related to work ability, as measured by the Work Ability Index (WAI). Cross-sectional studies showed a strong association between ERI-related work stress and reduction of work ability, with adjusted odds ratio ranging from 2.4 to 2.9 [14,15]. Recently, the relation of ERI and work ability was also supported by longitudinal findings from the First German Sociomedical of Employees [16]. While these studies looked on the effects of ERI on work ability, there is also a potential pathway from restricted work ability to ERI. Employees with poor work ability might experience higher costs of working because more effort may be required to perform the work [17]. In addition, restricted work ability makes a person less productive, at least if the workplace environment is not adjusted to the requirements of the worker’s functional restrictions [17]. Though labour agreements may at least temporarily protect the workers from loss of wages, it is likely that their loss of productivity will be accompanied by less esteem from supervisors and co-workers and fewer possibilities for job promotion. Thus, efforts are higher but the perceived rewards may be lower, resulting in effort-reward imbalance. However, studies that examine the effects of poor work ability on the balance of effort and reward are still lacking. Consequently, our study aimed to analyse the cross-sectional and longitudinal associations between ERI and work ability. We hypothesised that ERI would predict declined work ability as well as declined work ability would predict ERI.

## Methods

### Setting and participants

Data come from the Second German Sociomedical Panel of Employees (GSPE-II) [18]. The GSPE-II is a large-scale cohort study designed to identify environmental and personal risk factors affecting work ability and participation in working life. Overall, the gross survey sample included 3750 women and 3750 men aged 45–59 who were randomly selected from the register of the federal German Pension Insurance Fund (GPIF). The GPIF manages the pension insurance payments of white-collar employees in Germany and is therefore responsible for their rehabilitation claims and disability pensions. Baseline and follow-up data were collected by postal surveys in 2009 and 2010. Only those participants who explicitly consented to follow-up were contacted one year later. The study was approved by the data protection commissioner of the GPIF.

### Work Ability Index

Work ability was assessed using the German version of the Work Ability Index (WAI) [19], a health-related instrument assessing the degree to which workers consider their state of health adequate to cope with their job demands [19-21]. The questionnaire comprises the following items: (1) current work ability compared with lifetime best, (2) work ability in relation to the demands of the job, (3) number of current diseases diagnosed by a physician, (4) estimated work impairment due to diseases, (5) sick leave during the past year, (6) own prognosis of work ability two years from now and (7) mental resources. The WAI score varies from 7 to 49, with higher scores indicating better work ability. According to the authors, WAI scores can be grouped into poor (7–27), moderate (28–36), good (37–43) and excellent work ability (44–49) [19,21]. For our analyses, we aggregated poor and moderate work ability into one category (WAI < 37) [14,22-24]. Good and excellent work ability (WAI ≥ 37) was chosen as the reference group. The test-retest reliability of the WAI was previously shown to be consistent [25]. Moreover, several studies have confirmed that poor work ability is a risk factor for productivity loss at work, retirement intentions, long-term sickness absence, unemployment and early retirement [18,26-32].

### Effort-reward imbalance

ERI was measured using the white-collar version of the ERI questionnaire, which comprises 16 items [10]. Compared to the 17-item blue collar version, the effort scale of the white collar version omits the item concerning physically demanding work [10]. Five of the items assess the efforts invested, and eleven of the items assess the rewards obtained in terms of (a) esteem, (b) job security and (c) salary and job promotion. To mirror the notion of non-reciprocal exchange at work, the effort-reward ratio (ER ratio) was calculated as the ratio of both scales whereby the reward was multiplied by a correction factor to account for the different numbers of items in the numerator and the denominator. An ER ratio > 1 implies that efforts are higher than rewards and indicates ERI.

### Covariates

#### Socio-demographic data and socioeconomic situation

Gender and age were considered relevant socio-demographic variables. Educational level was added as an indicator of socioeconomic position. Education level was assessed according to the International Standard Classification of Education (ISCED-97) [33]. The ISCED-97 considers general, vocational and academic degrees on six levels. Levels of education were categorised as low (ISCED-97 < 4) or high (ISCED-97 ≥ 4).

#### Health-related behaviour

Physical exercise (less than two hours per week, at least two hours per week), cigarette smoking (smoker, non-smoker) and body mass index (BMI ≥ 25 kg/m^2^, BMI < 25 kg/m^2^) were selected as relevant indicators of health-related behaviour. These variables were aggregated into an index of health-related behaviour with scores of 0 to 3, where higher values represent a healthier lifestyle.

#### Physical demands

Physical demands were assessed using a list of four different occupational tasks (e.g. carrying heavy loads or working in physically awkward positions). This list was a shortened version of the instrument used in the German Cardiovascular Prevention Study [34]. Responders rated how demanding these tasks were using a 4-point scale ranging from not demanding to very strong. The four items were summed to yield an index score.

#### Psychological job demands and job control

To describe the psychosocial work environment, we assessed both dimensions of Karasek’s demand-control model (DCM): psychological job demands and job control [35]. Both dimensions of the DCM were operationalised using short proxy measures. Job control (e.g. sufficient opportunity to use one’s abilities) was assessed using four items, and psychological job demands (e.g. need to work fast or with conflicting instructions) were assessed using five items. Each item was rated on a 5-point scale. The scores of both multi-item measures were calculated by averaging the summed non-weighted item scores.

### Statistical analysis

Descriptive statistics were used to describe the characteristics of the study population. Confirmatory factor analyses (CFA) were performed to test whether the proposed factor models of the WAI and the ERI questionnaire fitted the data [36]. We calculated the Goodness of Fit Index (GFI), the Normed Fit Index (NFI) and the Comparative Fit Index (CFI). These indices yield values ranging from zero to one, whereby values close to one are indicative of good fit and those greater than 0.90 or, better, 0.95 generally indicate satisfactory fit [36]. To compare responders and non-responders, differences in continuous scores were tested with t tests, and differences in proportions were tested with chi-squared tests.

To examine associations between ERI and WAI, we estimated three sets of logistic regression models. Odds ratios (OR) were estimated as measures of association with corresponding 95% confidence intervals (CI). The first set of models was calculated to estimate cross-sectional associations. These models used the dichotomous WAI (WAI < 37 vs. WAI ≥ 37) as the dependent variable and ERI (ER ratio > 1 vs. ER ratio ≤ 1) as the independent variable (Models 1.1 to 1.3). The other sets of models were calculated to estimate longitudinal associations. One set of models used the dichotomous WAI (WAI < 37 vs. WAI ≥ 37) as the dependent variable and ERI (ER ratio > 1 vs. ER ratio ≤ 1) as the independent variable (Models 2.1 to 2.3). The other set of models used ERI (ER ratio > 1 vs. ER ratio ≤ 1) as the dependent variable and the dichotomous WAI (WAI < 37 vs. WAI ≥ 37) as the independent variable (Models 3.1 to 3.3). In our longitudinal analyses, the sample used to predict new cases of poor or moderate work ability was restricted to cases with good or excellent baseline work ability (Models 2.1 to 2.3). The sample used to predict new cases of ERI at follow-up, i.e. ER ratio > 1, was restricted to those with a baseline ER ratio ≤ 1 (Models 3.1 to 3.3). Firstly, crude associations between independent and dependent variables were estimated. The subsequent multivariate analyses were based on a three-model approach for each set of models: the first model adjusted the effects of the independent variable for age, gender and educational level, the second additionally adjusted for health-related behaviour, and the third model additionally adjusted for physical demands and indicators of the DCM. Adjustment was done to rule out associations between ERI and WAI and WAI and ERI resulting from influences of established risk factors. Test statistics were regarded as significant if the two-sided P value was less than 0.05. All calculations were performed with PASW Statistics 19 except the confirmatory factor analyses, which were done with AMOS 19.

## Results

### Participants

During the first survey in 2009, 7500 questionnaires were mailed, 26 of which were returned as undeliverable. A total of 2730 valid questionnaires were returned, corresponding to a response rate of 36.5%. Data on the gender and age of non-responders were obtained from the GPIF registers. Responders and non-responders did not differ in terms of age and gender (responders: mean age: 51.5 years, SD = 4.3; 51.1% female; non-responders: mean age: 51.3 years, SD = 4.3; 49.4% female). 2301 (84.3%) persons of the full baseline sample consented to a follow-up survey. Finally, 1636 (71.1%) contacted persons responded after one year.

**Figure 1:**
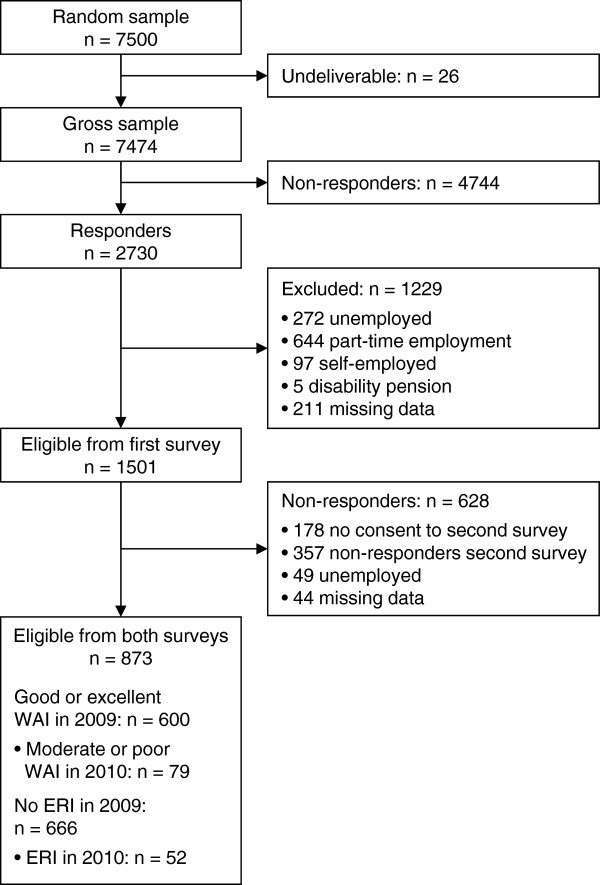
**Flow diagram of participants in the Second German Sociomedical Panel of Employees**. WAI, Work Ability Index; ERI, effort-reward imbalance.

Only first survey respondents with full-time employment were included in our analysis. Those who were unemployed (n = 272), working part-time (n = 644), self-employed (n = 97) or receiving disability pensions (n = 5) were excluded. Another 211 participants were excluded because of missing data for one of the baseline variables so that the final baseline sample comprised 1501 persons (Figure 1).

The mean age of the baseline sample was 51.1 years (SD = 4.2). 33.8% were female. The mean work ability score was 38.1 (SD = 7.5). Work ability was good or excellent in 65.2% of the participants and poor or moderate in 34.8%. 25.9% had an ER ratio > 1, indicating an imbalance of effort and reward (Table 1).

**Table 1:** Baseline sample characteristics

	**Mean (SD) or %**	**Median**	**IQR**	**Cronbach’s Alpha**
WAI, mean (SD)	38.1 (7.4)	39.5	34.0; 43.5	0.84
WAI				
Good or excellent, %	65.2			
Poor or moderate, %	34.8			
Gender: female, %	33.8			
Age, mean (SD)	51.1 (4.2)	51.0	47.0; 55.0	
Low educational level, %	34.8			
White-collar worker, %	97.4			
Exercise: at least two hours per week, %	46.6			
BMI ≥ 25, %	58.5			
Smoking, %	21.4			
Health-related behaviour (0–3), mean (SD)	1.7 (0.9)	2.0	1.0; 2.0	
Physical demands (0–12), mean (SD)	1.3 (2.0)	1.0	0.0; 2.0	0.68
Psychological job demands (0–4), mean (SD)	1.5 (0.9)	1.4	0.8; 2.2	0.82
Job control (0–4), mean (SD)	3.2 (0.7)	3.3	2.8; 3.8	0.79
Effort (5–25), mean (SD)	14.9 (4.5)	15.0	12.0; 18.0	0.84
Reward (11–55), mean (SD)	43.8 (8.7)	45.0	37.0; 52.0	0.89
ER ratio, mean (SD)	0.8 (0.4)	0.7	0.5; 1.0	
ERI: ER ratio > 1, %	25.9			

n = 1501; SD, standard deviation; IQR, interquartile range; WAI, Work Ability Index; BMI, body mass index; ER ratio, effort-reward ratio; ERI, effort-reward imbalance

CFA of the WAI dimensions showed that goodness of fit was about 0.95 (GFI = 0.956; NFI = 0.945; CFI = 0.948), indicating that the proposed one-factor model of the WAI fitted the sample data well. CFA of the ERI questionnaire data was also performed. The assumed model used effort and reward as correlating factors, where reward was further divided into three sub-components (esteem, job security, salary and job promotion). It fitted the data satisfactorily, with a goodness of fit about 0.90 (GFI = 0.896; NFI = 0.885; CFI = 0.893).

966 of the 1501 eligible baseline respondents provided follow-up data. 49 of whom were excluded as due to current unemployment, and 44 of whom were excluded because of missing WAI or ERI follow-up data. Follow-up respondents had better work ability, were less likely to smoke, had lower physical demands, reported less rewards and were less likely to experience ERI than non-responders. However, differences between responders and non-responders were small (Table 2).

**Table 2:** Baseline sample characteristics of follow-up responders and non-responders

	**Responders**	**Non-responders**	
	**n = 873**	**n = 628**	**p**
WAI, mean (SD)	38.7 (7.0)	37.2 (7.8)	< 0.001
WAI			
Good or excellent, %	68.7	60.4	0.001
Poor or moderate, %	31.3	39.6	
Gender: female, %	33.6	34.1	0.835
Age, mean (SD)	51.0 (4.0)	51.2 (4.3)	0.306
Low educational level, %	33.2	37.1	0.119
White-collar worker, %	97.8	96.8	0.226
Exercise: at least two hours per week, %	54.5	51.8	0.288
BMI ≥ 25, %	57.2	60.4	0.216
Smoking, %	19.6	23.9	0.045
Health-related behaviour (0–3), mean (SD)	1.7 (0.9)	1.6 (0.9)	0.301
Physical demands (0–12), mean (SD)	1.1 (1.7)	1.5 (2.2)	< 0.001
Psychological job demands (0–4), mean (SD)	1.5 (0.9)	1.5 (1.0)	0.528
Job control (0–4), mean (SD)	3.2 (0.7)	3.1 (0.7)	0.091
Effort (5–25), mean (SD)	14.8 (4.4)	15.1 (4.6)	0.229
Reward (11–55), mean (SD)	44.3 (8.7)	43.3 (8.8)	0.026
ER ratio, mean (SD)	0.8 (0.4)	0.8 (0.4)	0.083
ERI: ER ratio > 1, %	23.7	29.0	0.022

n = 1501; SD, standard deviation; WAI, Work Ability Index; BMI, body mass index; ER ratio, effort-reward ratio; ERI, effort-reward imbalance.

873 respondents had complete data sets from both surveys. The sample used for our longitudinal analyses was restricted to persons with good or excellent baseline work ability (n = 600; Model 2.1 to 2.3) when predicting new cases of poor or moderate work ability, and it was restricted to respondents with a baseline ER ratio ≤ 1 (n = 666; Model 3.1 to 3.3) when predicting new cases of ERI. The two samples were highly overlapped; 526 persons had good or excellent baseline work ability as well as a baseline ER ratio ≤ 1 (Table 3).

**Table 3:** Baseline characteristics of longitudinal samples

	**Predicting new cases of poor or moderate work ability**	**Predicting new cases of effort-reward imbalance**
	**n = 600**	**n = 666**
WAI, mean (SD)	42.5 (3.4)	40.4 (5.7)
WAI		
Good or excellent, %	100.0	79.0
Poor or moderate, %	-	21.0
Gender: female, %	30.0	33.3
Age, mean (SD)	50.7 (4.0)	51.0 (4.1)
Low educational level, %	27.3	31.8
White-collar worker, %	98.5	98.0
Exercise: at least two hours per week, %	53.0	53.8
BMI ≥ 25, %	55.3	55.0
Smoking, %	20.2	20.0
Health-related behaviour (0–3), mean (SD)	1.7 (0.9)	1.7 (0.9)
Physical demands (0–12), mean (SD)	0.7 (1.4)	0.8 (1.3)
Psychological job demands (0–4), mean (SD)	1.2 (0.8)	1.1 (0.8)
Job control (0–4), mean (SD)	3.4 (0.6)	3.3 (0.6)
Effort (5–25), mean (SD)	13.8 (4.1)	13.3 (3.6)
Reward (11–55), mean (SD)	47.0 (7.2)	47.7 (6.3)
ER ratio, mean (SD)	0.7 (0.3)	0.6 (0.2)
ERI: ER ratio > 1, %	12.3	-

SD, standard deviation; WAI, Work Ability Index; BMI, body mass index; ER ratio, effort-reward ratio; ERI, effort-reward imbalance.

After one year, we identified 79 (13.2%) new cases of poor or moderate work ability and 52 (7.8%) new cases of ERI, respectively (Figure 1).

### Cross-sectional association

All three models showed that ERI was associated with poor or moderate work ability (Table 4). Compared to persons without ERI, the odds of lower work ability were 6.1 and 6.0 times higher in Model 1.1 and 1.2. Although adjustment for other work-related characteristics (physical demands, psychological job demands and job control) reduced the odds of poor or moderate work ability, persons with ERI still had two times higher odds of poor or moderate work ability in Model 1.3 (OR = 1.980; 95% CI: 1.428 to 2.747). In the fully adjusted model (Model 1.3), lower work ability was also associated with female gender, higher age and lower educational level as well as higher physical demands, higher psychological job demands and lower job control.

**Table 4:** Cross-sectional association of effort-reward imbalance with poor or moderate work ability

	**Crude associations**		**Model 1.1**		**Model 1.2**		**Model 1.3**	
	**OR**	**95% CI**	**OR**	**95% CI**	**OR**	**95% CI**	**OR**	
Gender: female	1.756***	(1.407; 2.192)	1.804***	(1.405; 2.316)	1.850***	(1.438; 2.380)	1.672***	(1.270; 2.202)
Age	1.047***	(1.021; 1.074)	1.061***	(1.031; 1.092)	1.061***	(1.031; 1.092)	1.085***	(1.050; 1.120)
Low educational level	2.067***	(1.657; 2.577)	1.766***	(1.382; 2.256)	1.731***	(1.353; 2.214)	1.606**	(1.217; 2.120)
Health-related behaviour	0.858*	(0.759; 0.970)			0.888	(0.774; 1.019)	0.911	(0.782; 1.060)
Physical demands	1.601***	(1.487; 1.724)					1.291***	(1.192; 1.398)
Psychological job demands	2.661***	(2.331; 3.036)					1.880***	(1.584; 2.232)
Job control	0.315***	(0.263; 0.376)					0.409***	(0.334; 0.500)
ERI: ER ratio > 1	5.736***	(4.471; 7.359)	6.056***	(4.678; 7.840)	6.005***	(4.637; 7.776)	1.980***	(1.428; 2.747)
N-R^2^			0.227		0.230		0.404	

n = 1501; ERI, effort-reward imbalance; ER ratio, effort-reward ratio; OR, odds ratio; CI, confidence interval; N-R^2^, Nagelkerke-R^2^; *** p < 0.001; ** p < 0.01; * p < 0.05.

### Longitudinal associations

#### Predicting new cases of poor or moderate work ability

ERI predicted new cases of poor or moderate WAI in all three models (Table 5). Adjusted odds ratios ranged from roughly 3.4 in Model 2.1 to roughly 2.1 in Model 2.3 (OR = 2.093; 95% CI: 1.047 to 4.183). New cases of poor or moderate WAI were also associated with higher age, higher psychological job demands and lower job control. Better health-related behaviour significantly reduced the odds of poor or moderate work ability at follow-up.

**Table 5:** Predicting new cases of poor or moderate work ability

	**Crude associations**		**Model 2.1**		**Model 2.2**		**Model 2.3**	
	**OR**	**95% CI**	**OR**	**95% CI**	**OR**	**95% CI**	**OR**	
Gender: female	1.169	(0.705; 1.938)	1.130	(0.658; 1.942)	1.291	(0.742; 2.246)	1.238	(0.707; 2.170)
Age	1.045	(0.986; 1.108)	1.056	(0.993; 1.124)	1.058	(0.993; 1.127)	1.072*	(1.004; 1.144)
Low educational level	1.658*	(1.008; 2.728)	1.577	(0.931; 2.672)	1.543	(0.907; 2.624)	1.640	(0.943; 2.852)
Health-related behaviour	0.686**	(0.524; 0.898)			0.678**	(0.511; 0.901)	0.678**	(0.507; 0.905)
Physical demands	1.141	(0.993; 1.312)					1.007	(0.856; 1.185)
Psychological job demands	1.673***	(1.268; 2.207)					1.438*	(1.017; 2.033)
Job control	0.565**	(0.383; 0.834)					0.590*	(0.391; 0.892)
ERI: ER ratio > 1	3.197***	(1.800; 5.678)	3.374***	(1.881; 6.051)	3.334***	(1.852; 6.002)	2.093*	(1.047; 4.183)
N-R^2^			0.063		0.084		0.117	

n = 600; ERI, effort-reward imbalance; ER ratio, effort-reward ratio; OR, odds ratio; CI, confidence interval; N-R^2^, Nagelkerke-R^2^; *** p < 0.001; ** p < 0.01; * p < 0.05.

#### Predicting new cases of effort-reward imbalance

Poor or moderate work ability at baseline was a strong predictor of new cases of ERI at follow-up (Table 6). Persons with poor or moderate work ability at baseline had 3.2 times higher odds of ERI in Models 3.1 and 3.2 and 2.6 times higher odds in the fully adjusted model (Model 3.3) (OR = 2.573; 95% CI: 1.314 to 5.041). New cases of ERI were also predicted by higher psychological job demands.

**Table 6:** Predicting new cases of effort-reward imbalance

	**Crude associations**		**Model 3.1**		**Model 3.2**		**Model 3.3**	
	**OR**	**95% CI**	**OR**	**95% CI**	**OR**	**95% CI**	**OR**	
Gender: female	1.275	(0.711; 2.285)	1.066	(0.578; 1.966)	1.130	(0.609; 2.097)	1.099	(0.589; 2.051)
Age	0.962	(0.897; 1.033)	0.946	(0.878; 1.019)	0.944	(0.875; 1.017)	0.951	(0.881; 1.026)
Low educational level	1.256	(0.696; 2.265)	1.034	(0.550; 1.943)	0.984	(0.522; 1.856)	1.047	(0.545; 2.011)
Health-related behaviour	0.761	(0.550; 1.053)			0.753	(0.535; 1.061)	0.763	(0.537; 1.084)
Physical demands	1.229*	(1.047; 1.443)					1.109	(0.917; 1.341)
Psychological job demands	1.953***	(1.375; 2.773)					1.591*	(1.088; 2.327)
Job control	0.864	(0.556; 1.342)					1.172	(0.717; 1.917)
Poor or moderate WAI	3.082***	(1.716; 5.537)	3.235***	(1.757; 5.958)	3.189***	(1.728; 5.887)	2.573**	(1.314; 5.041)
N-R^2^			0.055		0.064		0.094	

n = 666; WAI, Work Ability Index; OR, odds ratio; CI, confidence interval; N-R^2^, Nagelkerke-R^2^; *** p < 0.001; ** p < 0.01; * p < 0.05.

## Discussion

The aim of our study was to analyse cross-sectional and longitudinal associations of ERI and WAI. After adjustment for age, gender, educational level, health-related behaviour and other factors of the work environment (physical demands, job control and psychological job demands), ERI had an effect on WAI in the cross-sectional and longitudinal analyses. These results are in line with recent cross-sectional [14,15,37] and longitudinal findings [16] and confirm that reciprocity and fairness at work, as operationalised by Siegrist’s ERI model, have a relevant impact on work ability independent of and above that of other known explanatory variables. However, it is notable that our findings also demonstrate that low work ability negatively affects the balance of effort and reward. This indicates that ERI might be a risk factor that mediates the transition from poor work ability to exit from paid employment and health-related early retirement [18,26-32]. Furthermore, there seems to be a downward spiral of ERI affecting work ability that again might intensify the perception of an imbalanced relation of effort and reward.

Besides the adverse effect of ERI on WAI, our analyses showed that higher age, poorer health-related behaviour, higher psychological job demands and lower job control also predicted new cases of poor or moderate WAI. This is line with the review by van den Berg et al. [22], which presented consistent evidence supporting the association of poor work ability primarily with older age, lack of leisure-time activity and obesity but also with high mental work demands and lack of autonomy. In contrast to the aforementioned review [22], we could not establish a prospective association between physical demands and work ability, even though the cross-sectional association was relatively strong.

Despite a degree of concordance with the review of van den Berg [22], our study suffers from several limitations.

Firstly, the response rate of the first survey was rather low. Other authors have described such a response rate as reasonable for an anonymous survey in the working population [38]. However, we cannot rule out the possibility of bias from selective participation. Although our baseline responders and non-responders did not differ in terms of gender and age, we were unable to investigate the characteristics of the baseline non-responders in depth.

Secondly, our analyses of follow-up responders and non-responders indicated selective follow-up participation suggestive of a healthy worker effect.

Thirdly, as our results were based on an older, full-time employed white-collar sample, this certainly constrains the generalisation of our findings.

Fourthly, the gap between baseline and follow-up measurement was only one year, and baseline measurements were restricted to single-point measurements of the explanatory variables. As the time of exposure is relevant to the establishment of causal relations, a longer follow-up and measures of continuous exposure could provide a better understanding of the associations between ERI and work ability.

Fifthly, as both ERI and work ability were measured by self-report questionnaires, a tendency to respond negatively could have inflated the cross-sectional association. Some authors therefore propose to adjust regression models for negative affectivity when analysing associations between self-reported work stressors and measures of health [39-41], whereas others strongly advise against adjusting for negative affectivity [42]. We did not adjust for possible response bias due to negative affectivity. Indeed, a recent simulation study suggested that negative affectivity can affect associations of ERI and health-related outcomes even if mean scores are only slightly changed. However, substantial effects on the association are only plausible if a large proportion of participants and their questionnaire answers are influenced by negative affectivity [43]. Moreover, we assume that negative affectivity did not affect our longitudinal analyses as these analyses were restricted to persons with good work ability and persons without effort-reward imbalance, respectively.

Sixthly, psychological job demands were strongly correlated with the binary ERI variable. This might result in overadjustment, with ERI as the dependent variable, and multicollinearity, with WAI as the dependent and ERI as the independent variable. We dropped psychological job demands from our final models and repeated the parameter estimations. Standard errors were slightly reduced. Estimates of the effect of ERI on work ability increased roughly 2-fold in the cross-sectional and longitudinal analyses. The estimate of the longitudinal effect of poor or moderate work ability on ERI was only slightly affected in the longitudinal analysis.

Seventhly, our study presents findings within the context of the German system of social security, employees’ rights and employers’ duties. However, as was recently shown for the associations between job insecurity and health-related outcomes, results may differ between welfare regimes [3].

Nonetheless, these limitations are balanced by several strengths. Firstly, participants were recruited by random sampling. Secondly, we could refer to a relatively large sample for our analyses. Thirdly, our analyses were performed using a longitudinal design. Fourthly, the analyses were restricted to cases with good or excellent baseline work ability and cases without baseline ERI, respectively, in order to predict new cases of adverse events.

Our results concerning the effect of ERI on work ability indicate that an adequate effort-reward balance at work is a crucial dimension of healthy work. In this context, the ERI model offers options to promote work ability at the individual, interpersonal and organisational level [16,44]. While individual-level interventions focus on coping with the existing stressors (e.g. reducing overcommitment in order to rebalance efforts and rewards), other interventions can be designed to modify stressors at the interpersonal or organisational level. For instance, Bourbonnais et al. [45] described a participatory intervention approach in an acute care hospital in Canada. Following the concepts of German health circles, a multi-professional team of staff members and researchers identified 56 intervention targets and developed proposals for solutions. A controlled trial demonstrated that ERI decreased after one and three years in the intervention group compared to the controls and showed that most of the recommended solutions could be permanently implemented [46].

Although there is a strong body of evidence concerning the impact of work ability on productivity loss at work, retirement intentions, long-term sickness absence, unemployment and early retirement [18,26-32], there is less research regarding its more proximal consequences for work environment, work demands and quality of work. Cross-sectional studies that investigated associations between work characteristics and work ability were mostly interpreted unidirectionally in terms of work characteristics affecting work ability. Our longitudinal analyses show that causal relations also act conversely. This indicates that occupational health services for persons with poor work ability must not be restricted to the workers’ physical requirements. Employees also need support that addresses their psychosocial demands, especially esteem and security, in order to prevent effort-reward imbalance.

## Conclusions

ERI at work was associated with a higher risk of poor or moderate work ability after one year. Moreover, persons with poor or moderate work ability were more prone to develop ERI than persons with good or excellent work ability. Interventions at the individual, interpersonal and organisational level designed to enable workers to cope with ERI or which address indicators of ERI directly could promote the maintenance of work ability. Integration management and occupational health services for persons with poor work ability should also consider their psychosocial demands.

## Competing interests

The authors declare that they have no competing interests.

## Authors’ contributions

MB was responsible for data management, analysed the data and drafted the manuscript. FMR developed the design of the Second German Sociomedical Panel of Employees and helped to analyse the data. CG helped to draft the manuscript. All authors read and approved the final manuscript.

## Pre-publication history

The pre-publication history for this paper can be accessed here:

http://www.biomedcentral.com/1471-2458/12/875/prepub
